# Aerobic Exercise During Advance Stage of Uncontrolled Arterial Hypertension

**DOI:** 10.3389/fphys.2021.675778

**Published:** 2021-06-03

**Authors:** Luana U. Pagan, Mariana J. Gomes, Ricardo L. Damatto, Aline R. R. Lima, Marcelo D. M. Cezar, Felipe C. Damatto, David R. A. Reyes, Dijon H. S. Campos, Tulio M. M. Caldonazo, Bertha F. Polegato, Denise C. Fernandes, Francisco R. Laurindo, Ana A. H. Fernandes, Ana Lloret, Antonio C. Cicogna, Marina P. Okoshi, Katashi Okoshi

**Affiliations:** ^1^Botucatu Medical School, UNESP, Sao Paulo State University, Botucatu, Brazil; ^2^Itapeva Social and Agrarian Sciences College, FAIT, Itapeva, Brazil; ^3^Department of Cardiopneumology, Medical School, University of Sao Paulo (USP), São Paulo, Brazil; ^4^Institute of Biosciences, Sao Paulo State University (UNESP), Botucatu, Brazil; ^5^Freshage Research Group, Department of Physiology, University of Valencia, CIBERFES, INCLIVA, Valencia, Spain

**Keywords:** heart, physical exercise, spontaneously hypertensive rats, hypertension, myocardium function

## Abstract

**Aim:**

To evaluate the influence of physical training on myocardial function, oxidative stress, energy metabolism, and MAPKs and NF-κB signaling pathways in spontaneously hypertensive rats (SHR), at advanced stage of arterial hypertension, which precedes heart failure development.

**Methods:**

We studied four experimental groups: normotensive Wistar rats (W, *n* = 27), trained W (W-EX, *n* = 31), SHR (*n* = 27), and exercised SHR (SHR-EX, *n* = 32). At 13 months old, the exercise groups underwent treadmill exercise 5 days a week for 4 months. *In vitro* myocardial function was analyzed in left ventricular (LV) papillary muscle preparations. Antioxidant enzyme activity and energy metabolism were assessed by spectrophotometry. Nicotinamide adenine dinucleotide phosphate (NADPH) oxidase activity was analyzed by lucigenin reduction and protein expression by Western blot. Statistical analyzes: ANOVA and Tukey or Kruskal–Wallis and Dunn tests.

**Results:**

SHR-EX had a lower frequency of heart failure features than SHR. Myocardial function and antioxidant enzyme activity were better in SHR-EX than SHR. Lipid hydroperoxide concentration, and phosphorylated JNK and total IkB protein expression were higher in hypertensive than control groups. Malondialdehyde, NADPH oxidase activity, total JNK, phosphorylated p38, phosphorylated and total p65 NF-κB, and phosphorylated IkB did not differ between groups. Protein expression from total p38, and total and phosphorylated ERK were higher in SHR than W. Lactate dehydrogenase and phosphorylated ERK were lower and citrate synthase and β-hydroxyacyldehydrogenase were higher in SHR-EX than SHR.

**Conclusion:**

Exercise improves physical capacity, myocardial function, and antioxidant enzyme activity; reduces the frequency of heart failure features and ERK phosphorylation; and normalizes energy metabolism in SHR.

## Introduction

Systemic arterial hypertension is one of the most important public health problems due to a high prevalence and relevant clinical consequences (Virani et al., 2021). Uncontrolled arterial hypertension is a major cause of cardiac remodeling; it is defined as myocardial gene, molecular, cellular and interstitial alterations manifesting clinically as changes in heart size, shape, and function (Cohn et al., 2000). Hallmark cardiac complications of arterial hypertension include left ventricular (LV) hypertrophy, which evolves with diastolic and systolic ventricular dysfunction, arrhythmias and heart failure.

Increased oxidative stress is involved in the pathogenesis of cardiac remodeling (Rababa’h et al., 2018). Oxidative stress follows an imbalance between reactive oxygen species (ROS) production and antioxidant defense. The nicotinamide adenine dinucleotide phosphate (NADPH) oxidase complex is an important source of ROS in the heart (Touyz et al., 2020). Increased NADPH oxidase activity and expression has been associated with LV hypertrophy in pressure overload models (Li et al., 2002; Zhao et al., 2020). The main cellular defenses against increased ROS include enzymes such as superoxide dismutase (SOD), catalase (CAT), and glutathione peroxidase (GSH-Px) (D’Oria et al., 2020). Increased oxidative stress activates the signaling pathways mitogen-activated protein kinases (MAPK) and nuclear factor-kappa B (NF-κB), which modulates several cellular functions such as gene expression, apoptosis, and hypertrophy (Tsutsui et al., 2011; Touyz et al., 2020).

Physical exercise attenuates cardiovascular risk factors (Lin et al., 2020). Studies on the effects of exercise on cardiac remodeling have shown an improvement in contractility, prevention of apoptosis, and attenuation of myocardial fibrosis and LV adverse remodeling (Huang et al., 2012; Locatelli et al., 2017). However, the effects of physical exercise during persistent pressure overload are not completely understood and both beneficial and adverse effects have been described (Schultz et al., 2007; da Costa Rebelo et al., 2012; Locatelli et al., 2017; Schreckenberg et al., 2017). We have previously observed that low intensity aerobic exercise improves diastolic function by attenuating myocardial fibrosis and metalloproteinase-2 activity (Pagan et al., 2015, 2019).

The spontaneously hypertensive rat (SHR) is an experimental model of genetic hypertension. The SHR presents early systemic arterial hypertension and LV hypertrophy which evolves to heart failure during maturity and senescence. As cardiac failure development is slow and similar to human hypertensive cardiomyopathy, the model has been widely used to study cardiac remodeling and heart failure (Bing et al., 1995; Damatto et al., 2016). In this study we evaluated the influence of physical exercise training on myocardial function, oxidative stress, energy metabolism, and the MAPK and NF-κB signaling pathways in SHR at an advanced stage of arterial hypertension, which precedes the development of heart failure.

## Materials and Methods

We studied normotensive male Wistar rats purchased from the Central Animal House at Botucatu Medical School, UNESP, and male SHR from the Central Biotherm of the Biomedical Sciences Institute, University of Sao Paulo, USP. All animals were housed in a room under temperature control at 24°C and kept on a 12-h light/dark cycle. Food and water were supplied *ad libitum*. The study protocol was approved by the Botucatu Medical School Animal Experimentation Ethics Committee, UNESP, São Paulo, Brazil.

Thirteen-month-old rats were divided into four groups: Wistar (W, *n* = 27); exercised Wistar (W-EX, *n* = 31); SHR (SHR, *n* = 27); and exercised SHR (SHR-EX, *n* = 32) groups. An initial echocardiogram was performed to ensure homogeneity between groups (results not shown). We assessed systolic blood pressure and maximum physical capacity before and at the end of the exercise period. Systolic arterial pressure was measured by tail-cuff plethysmography (Narco Bio-System^®^, model 709-0610, International Biomedical, Inc., United States) (Cicogna et al., 2000; Cezar et al., 2015). Maximum exercise capacity was assessed on a graded treadmill. Prior to evaluation, rats underwent an adaptation period of 10 min/day for 1 week; speed started at 6 m/min and was increased by 3 m/min every 3 min until rats were unable to run (Carvalho et al., 2005; Gomes et al., 2016). Rats were considered exhausted when they refused to run even after sound stimulation or were unable to coordinate steps. Maximum velocity was recorded and total distance calculated.

Physical training protocol was based in studies evaluating aged SHR (Emter et al., 2005; Pagan et al., 2015). Exercise was initiated at 13 months of age and continued for 16 weeks. During an adaptive period, exercise velocity was slowly increased from 5 to 17 m/min, and exercise duration from 10 to 45 min (Table 1). In the first 2 weeks of training, the animals were subjected to a low-voltage electrical stimulation to start exercise.

**TABLE 1:** Exercise protocol.

Weeks	Speed (m/min)	Duration (min)	Weekly exercise volume (m)
1st	5	10	250
2nd	7.5	16	600
3rd	10	22	1,100
4th	13	26	1,690
5th	15	36	2,700
6th	17	45	3,375

*Weekly exercise volume, run distance per week; m, meter; min, minute; %, percentage of maximum speed in the initial exercise test.*

### Myocardial Functional Study

Intrinsic myocardial contractile performance was evaluated using isolated LV papillary muscle prepared as previously described (Matsubara et al., 1997; Okoshi et al., 2001, 2004). Rats were anesthetized with pentobarbital sodium, 50 mg/kg, intraperitoneal, and decapitated. After removing hearts, the LV anterior or posterior papillary muscle was dissected free and placed in a chamber containing Krebs–Henseleit solution at 28°C and oxygenated with a mixture of 95 % O_2_ and 5 % CO_2_, pH 7.38. A Kyowa model 120T-20B transducer was used to register force and a lever system was used to adjust muscle length. Preparations were stimulated 12 times/min by electrodes delivering 5-ms pulses at a voltage 10 % above threshold. After a 60 min period of isotonic contractions, muscles were loaded to contract isometrically and stretched to the apices of their length-tension curves (Lmax). Following a 15 min period of stable isometric contraction, one isometric contraction was recorded for later analysis. The following parameters were measured from isometric contraction: developed tension [DT, g/mm (Cohn et al., 2000)], resting tension (RT, g/mm^2^), maximum rate of tension development (+dT/dt, g/mm^2^/s), and maximum rate of tension decrease. To evaluate myocardial contractile reserve, mechanical performance was analyzed at basal condition and after the following positive inotropic stimulation: post-rest contractions (10, 30, and 60 s), increased extracellular Ca^2+^ concentration (external calcium concentrations of 0.625, 1.25, and 2.5 mM), and addition of β-adrenergic agonist isoproterenol (10^–8^, 10^–7^, and 10−6 M) to the nutrient solution (Rosa et al., 2016). Papillary muscle cross-sectional area was calculated from muscle weight and length by assuming cylindrical uniformity and a specific gravity of 1.0. All force data were normalized for muscle cross-sectional area.

### Antioxidant Enzyme Activities, Lipid Hydroperoxide Concentration, and Energy Metabolism

Left ventricular samples (∼200 mg) were homogenized in 2 mL of cold 0.1 M phosphate buffer, pH 7.0 (Martinez et al., 2015). Tissue homogenates were centrifuged at 10,000 *g*, for 15 min at 4°C, and the supernatant assayed for total protein, lipid hydroperoxide (Rosa et al., 2016), and GSH-Px (E.C.1.11.1.9), CAT (E.C.1.11.1.6.), and SOD (E.C.1.15.1.1.) activities by spectrophotometry (Gimenes et al., 2015). Supernatant was also used for evaluating cardiac energy metabolism by measuring β-hydroxyacyl dehydrogenase (β-HAD), citrate synthase, and dehydrogenase activities as previously described (Seiva et al., 2009).

### Serum and Myocardial Malondialdehyde Concentration

Systemic lipid peroxidation was assessed by measuring serum malondialdehyde (MDA) concentration by high performance liquid chromatography (HPLC), as previously reported (Nielsen et al., 1997; Reyes et al., 2017). For myocardial MDA evaluation, tissue fragments (≅50 mg) were homogenized in 50 mM KPi with 1 mM EDTA buffer, pH 7.4. Samples were centrifuged at 500 *g* for 5 min at 4°C and the supernatant removed. After quantifying protein, 25 μL of the supernatant was incubated for 60 min at 95°C in 500 μL of 2 M anhydrous sodium acetate buffer, pH 3.5, with 0.2 % thiobarbituric acid (TBA). Then, 500 μL of 50 mM KH_2_PO_4_ buffer, pH 6.8, was added to the sample and centrifuged at 13,000 *g* for 5 min at 4°C. After, 200 μL of the supernatant was removed and 200 μL of the 50 mM KH_2_PO_4_ buffer was added; the mixture was analyzed by HPLC (Buege and Aust, 1978).

### Myocardial NADPH Oxidase Activity

Left ventricular fragments (∼200mg) were washed in PBS to remove blood and homogenized in 1mL of ice-cold lysis buffer containing 50 mM Tris (pH 7.4) (Gomes et al., 2016). NADPH oxidase activity was evaluated in membrane-enriched cellular fraction by reduction of lucigenin detected by luminometer as previously described (Laurindo et al., 2008).

### Western Blotting

Protein levels were analyzed by Western blotting as previously described (Lima et al., 2014; Martinez et al., 2016) using specific antibodies (Santa Cruz Biotechnology, Santa Cruz, CA, United States): anti- total JNK1/2 (sc-137019), p-JNK (sc-6254), total p38-MAPK (sc-7972), p-p38-MAPK (sc-17852), total ERK 1 (sc-93), and p-ERK1/2 (sc-16982). Protein levels were normalized to GAPDH (6C5 sc-32233). Myocardial protein was extracted using RIPA buffer containing protease and phosphatase inhibitors; supernatant protein content was quantified by the Bradford method. Samples were separated on a polyacrylamide gel and transferred to a nitrocellulose membrane. After blockade, membranes were incubated with the primary antibodies. The membranes were then washed with TBS and Tween 20 and incubated with secondary peroxidase-conjugated antibodies. Super Signal^®^ West Pico Chemiluminescent Substrate (Pierce Protein Research Products, Rockford, IL, United States) was used to detect bound antibodies. The membrane was then stripped (Restore Western Blot Stripping Buffer, Pierce Protein Research Products, Rockford, IL, United States) to remove previous antibody and incubated with anti-GAPDH antibody.

### Statistical Analysis

Data are expressed as means ± standard deviation or medians and percentiles. Normality was assessed by the Shapiro–Wilk test. Comparisons between groups were performed by analysis of variance (ANOVA) for a 2 × 2 factorial design followed by the Tukey *post hoc* test or Kruskal–Wallis and Dunn’s test; comparisons of interest: W-EX vs. W, SHR vs. W, SHR-EX vs. SHR, and SHR-EX vs. W-EX. Mortality rate and heart failure feature frequencies were assessed by the Goodman test. Significance level was set at 5%.

## Results

**FIGURE 1:**
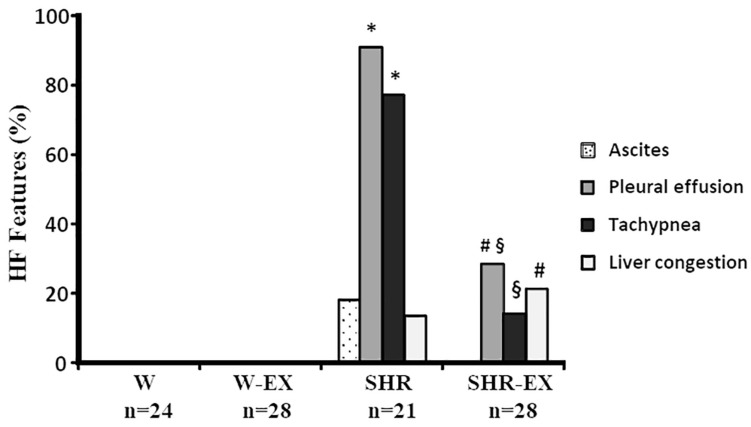
Heart failure (HF) feature frequencies. W: Wistar rats; W-EX: exercised Wistar rats; SHR: spontaneously hypertensive rats; SHR-EX: exercised SHR. Goodman test; ^∗^*p* < 0.05 vs. W; #*p* < 0.05 vs. W-EX; §*p* < 0.05 vs. SHR.

Three rats from each normotensive group, six from SHR, and four from SHR-EX died during the experiment (*p* > 0.05). Physical exercise did not change blood pressure (final systolic blood pressure: W 119 ± 11.0; W-EX 114 ± 8.57; SHR 194 ± 21.6; SHR-EX 188 ± 12.2 mmHg; *p* < 0.05 SHR and SHR-EX vs. their respective controls). Exercise increased run distance and time in the maximum exercise test (Table 2). Frequency of HF features is shown in Figure 1. Both W and W-EX had no HF features. SHR had a higher frequency of rats with pleural effusion and tachypnea than W and SHR-EX groups. SHR-EX presented a higher percentage of pleural effusion and hepatic congestion than W-EX.

**TABLE 2:** Maximum exercise test.

		W (*n* = 16)	W-EX (*n* = 16)	SHR (*n* = 22)	SHR-EX (*n* = 28)
Time (min)	Initial test	19.0 ± 1.6	16.4 ± 1.9	22.0 ± 2.3*	21.3 ± 2.0^#^
	Final test	17.0 ± 2.1	28.5 ± 2.3*	22.0 ± 2.3*	32.5 ± 2.6^#§^
Distance (m)	Initial test	156 ± 27	161 ± 32	276 ± 50*	256 ± 42^#^
	Final test	175 ± 37	449 ± 50*	250 ± 37*	568 ± 86^#§^

Data as mean ± standard deviation. W, Wistar rats; W-EX, exercised Wistar rats; SHR, spontaneously hypertensive rats; SHR-EX, exercised SHR. ANOVA for a 2 × 2 factorial design and Tukey; *p < 0.05 vs. W; #*p* < 0.05 vs. W-EX; §*p* < 0.05 vs. SHR.

Myocardial function results evaluated in isolated papillary muscles at basal condition are shown in Table 3. SHR had higher resting tension and –dT/dt than W. All variables were higher in SHR-EX than W-EX, and +dT/dt was higher in SHR-EX than SHR. Table 4 shows myocardial function after 10-, 30-, and 60-s post-resting contractions. SHR-EX had higher DT, RT, and -dT/dt than W-EX after all pauses. RT was higher in SHR than W after the 30- and 60-s pauses. There were no differences between SHR-EX and SHR. Table 5 shows myocardial function after changes in extracellular calcium concentration. SHR had higher DT, RT, and -dT/dt than W at 0.625 and 1.25 mM calcium concentrations. At 2.5 mM extracellular calcium concentration, only RT was higher in SHR than W. SHR-EX had higher DT, RT, and -dT/dt than W-EX at all calcium concentrations, and higher DT and +dT/dt than SHR at 0.625 mM calcium concentration. Table 6 shows functional evaluation after isoproterenol stimulation. At basal condition, all parameters were higher in SHR-EX than W-EX, and +dT/dt was higher in SHR-EX than SHR. RT was higher in both SHR and SHR-EX than their controls in all contractions. SHR-EX had higher DT than W-EX after 10^–8^ M isoproterenol.

**TABLE 3:** Basal papillary muscle functional data.

	W (*n* = 14)	W-EX (*n* = 13)	SHR (*n* = 18)	SHR-EX (*n* = 17)
CSA (mm^2^)	1.26 ± 0.15	1.24 ± 0.15	1.33 ± 0.17	1.24 ± 0.19
DT (g/mm^2^)	5.02 ± 1.41	4.84 ± 1.10	5.77 ± 1.32	6.35 ± 0.82^#^
RT (g/mm^2^)	0.57 ± 0.17	0.52 ± 0.09	0.78 ± 0.15*	0.79 ± 0.19^#^
+dT/dt (g/mm^2^/s)	53.3 ± 16.8	50.4 ± 11.5	52.2 ± 12.8	63.4 ± 9.78^#§^
–dT/dt (g/mm^2^/s)	20.2 ± 4.74	19.6 ± 3.49	23.2 ± 4.41*	24.7 ± 3.30^#^

Data as mean ± standard deviation. W, Wistar rats; W-EX, exercised Wistar rats; SHR, spontaneously hypertensive rats; SHR-EX, exercised SHR; CSA, papillary muscle cross-sectional area; DT, developed tension; RT, resting tension; +dT/dt, maximum rate of tension development; –dT/dt, maximum rate of tension decrease. ANOVA for a 2 × 2 factorial design and Tukey; *p < 0.05 vs. W; #*p* < 0.05 vs. W-EX; §*p* < 0.05 vs. SHR.

**TABLE 4:** Papillary muscle function after post-resting contraction.

	Groups	PP 10 s	PP 30 s	PP 60 s
DT (g/mm^2^)	W	5.94 ± 1.53	6.60 ± 1.49	6.81 ± 1.52
	W-EX	5.82 ± 1.12	6.47 ± 1.21	6.65 ± 1.25
	SHR	6.49 ± 1.38	7.05 ± 1.45	7.26 ± 1.48
	SHR-EX	7.30 ± 1.53^#^	7.80 ± 1.53^#^	7.98 ± 1.60^#^
RT (g/mm^2^)	W	0.56 ± 0.17	0.56 ± 0.16	0.55 ± 0.16
	W-EX	0.52 ± 0.08	0.52 ± 0.11	0.51 ± 0.12
	SHR	0.82 ± 0.19	0.80 ± 0.19*	0.77 ± 0.19*
	SHR-EX	0.84 ± 0.24^#^	0.85 ± 0.25^#^	0.82 ± 0.23^#^
+dT/dt (g/mm^2^/s)	W	63.0 ± 18.7	69.7 ± 17.5	72.2 ± 17.5
	W-EX	61.6 ± 12.6	69.5 ± 14.4	71.3 ± 14.7
	SHR	58.7 ± 14.4	65.0 ± 15.6	67.1 ± 15.5
	SHR-EX	71.6 ± 14.5	77.2 ± 16.5	78.6 ± 18.0
-dT/dt (g/mm^2^/s)	W	21.5 ± 4.36	23.2 ± 4.47	23.6 ± 4.25
	W-EX	21.8 ± 3.28	22.8 ± 3.37	23.3 ± 3.08
	SHR	23.8 ± 4.01	24.7 ± 3.96	25.7 ± 4.28
	SHR-EX	25.5 ± 3.60^#^	26.0 ± 3.91^#^	26.9 ± 4.40^#^

Data as mean ± standard deviation. W, Wistar rats; W-EX, exercised Wistar rats; SHR, spontaneously hypertensive rats; SHR-EX, exercised SHR; DT, developed tension; RT, resting tension; +dT/dt, maximum rate of tension development; –dT/dt, maximum rate of tension decreases. ANOVA for a 2 × 2 factorial design and Tukey; *p < 0.05 vs. W; #*p* < 0.05 vs. W-EX.

**TABLE 5:** Papillary muscle function after changes in extracellular calcium concentration.

	Groups	0.625 mM	1.25 mM	2.5 Mm
DT (g/mm^2^)	W	3.36 ± 0.82	5.27 ± 0.98	6.13 ± 1.38
	W-EX	3.34 ± 0.77	5.14 ± 0.83	5.88 ± 1.10
	SHR	4.40 ± 1.27*	6.15 ± 1.55*	6.58 ± 1.33
	SHR-EX	5.16 ± 0.97^#§^	6.62 ± 1.26^#^	6.87 ± 1.33^#^
RT (g/mm^2^)	W	0.49 ± 0.14	0.49 ± 0.14	0.49 ± 0.13
	W-EX	0.46 ± 0.10	0.45 ± 0.09	0.47 ± 0.09
	SHR	0.71 ± 0.15*	0.70 ± 0.16*	0.72 ± 0.19*
	SHR-EX	0.74 ± 0.23^#^	0.75 ± 0.23^#^	0.80 ± 0.25^#^
+dT/dt (g/mm^2^/s)	W	35.0 ± 9.12	56.7 ± 12.5	69.3 ± 16.9
	W-EX	35.2 ± 6.42	55.7 ± 8.69	66.2 ± 14.1
	SHR	38.5 ± 10.4	58.5 ± 16.7	63.8 ± 14.6
	SHR-EX	48.8 ± 9.53^#§^	66.5 ± 12.7	71.9 ± 15.2
-dT/dt (g/mm^2^/s)	W	15.8 ± 3.50	22.2 ± 3.61	23.4 ± 4.68
	W-EX	15.1 ± 2.26	21.6 ± 2.42	22.7 ± 3.43
	SHR	20.0 ± 5.40*	25.5 ± 5.29*	26.0 ± 4.22
	SHR-EX	22.4 ± 3.45^#^	26.2 ± 3.99^#^	26.4 ± 4.05^#^

Data as mean ± standard deviation. W, Wistar rats; W-EX, exercised Wistar rats; SHR, spontaneously hypertensive rats; SHR-EX, exercised SHR; DT, developed tension; RT, resting tension; +dT/dt, maximum rate of tension development; –dT/dt, maximum rate of tension decrease. ANOVA for a 2 × 2 factorial design and Tukey; *p < 0.05 vs. W; #*p* < 0.05 vs W-EX; §*p* < 0.05 vs. SHR.

**TABLE 6:** Papillary muscle function after isoproterenol (ISO) stimulation.

	Groups	Basal	10^–8^ M ISO	10^–7^ M ISO	10^–6^ M ISO
DT (g/mm^2^)	W	5.00 ± 1.00	5.29 ± 1.08	5.50 ± 1.09	5.65 ± 1.11
	W-EX	4.85 ± 0.82	5.14 ± 0.84	5.39 ± 1.00	5.59 ± 1.06
	SHR	5.60 ± 1.32	5.67 ± 1.37	5.71 ± 1.29	5.57 ± 1.17
	SHR-EX	6.23 ± 1.20^#^	6.34 ± 1.33^#^	6.33 ± 1.51	6.31 ± 1.63
RT (g/mm^2^)	W	0.49 ± 0.14	0.45 ± 0.14	0.46 ± 0.19	0.41 ± 0.13
	W-EX	0.49 ± 0.12	0.44 ± 0.13	0.42 ± 0.12	0.40 ± 0.11
	SHR	0.78 ± 0.18*	0.73 ± 0.17*	0.71 ± 0.17*	0.68 ± 0.17*
	SHR-EX	0.85 ± 0.24^#^	0.81 ± 0.23^#^	0.78 ± 0.23^#^	0.76 ± 0.22^#^
+dT/dt (g/mm^2^/s)	W	53.2 ± 12.4	60.6 ± 13.8	67.1 ± 13.8	73.1 ± 16.9
	W-EX	52.3 ± 8.32	57.6 ± 8.83	65.6 ± 11.4	70.9 ± 11.9
	SHR	51.6 ± 11.9	56.9 ± 14.1	62.3 ± 15.4	64.3 ± 18.7
	SHR-EX	62.5 ± 12.4^#§^	66.6 ± 13.5	71.8 ± 16.1	76.1 ± 18.7
-dT/dt (g/mm^2^/s)	W	21.0 ± 3.86	24.8 ± 4.35	31.9 ± 5.40	40.7 ± 7.05
	W-EX	20.2 ± 2.46	23.5 ± 2.68	30.4 ± 3.41	40.0 ± 4.75
	SHR	23.7 ± 5.23	24.8 ± 5.31	32.2 ± 6.72	41.4 ± 8.15
	SHR-EX	24.6 ± 4.02^#^	25.9 ± 3.95	31.0 ± 4.95	41.7 ± 6.69

Data as mean ± standard deviation. W, Wistar rats; W-EX, exercised Wistar rats; SHR, spontaneously hypertensive rats; SHR-EX, exercised SHR; DT, developed tension; RT, resting tension; +dT/dt, maximum rate of tension development; –dT/dt, maximum rate of tension decrease. ANOVA for a 2 × 2 factorial design and Tukey; *p < 0.05 vs. W; #*p* < 0.05 vs. W-EX; §*p* < 0.05 vs. SHR.

**FIGURE 2:**
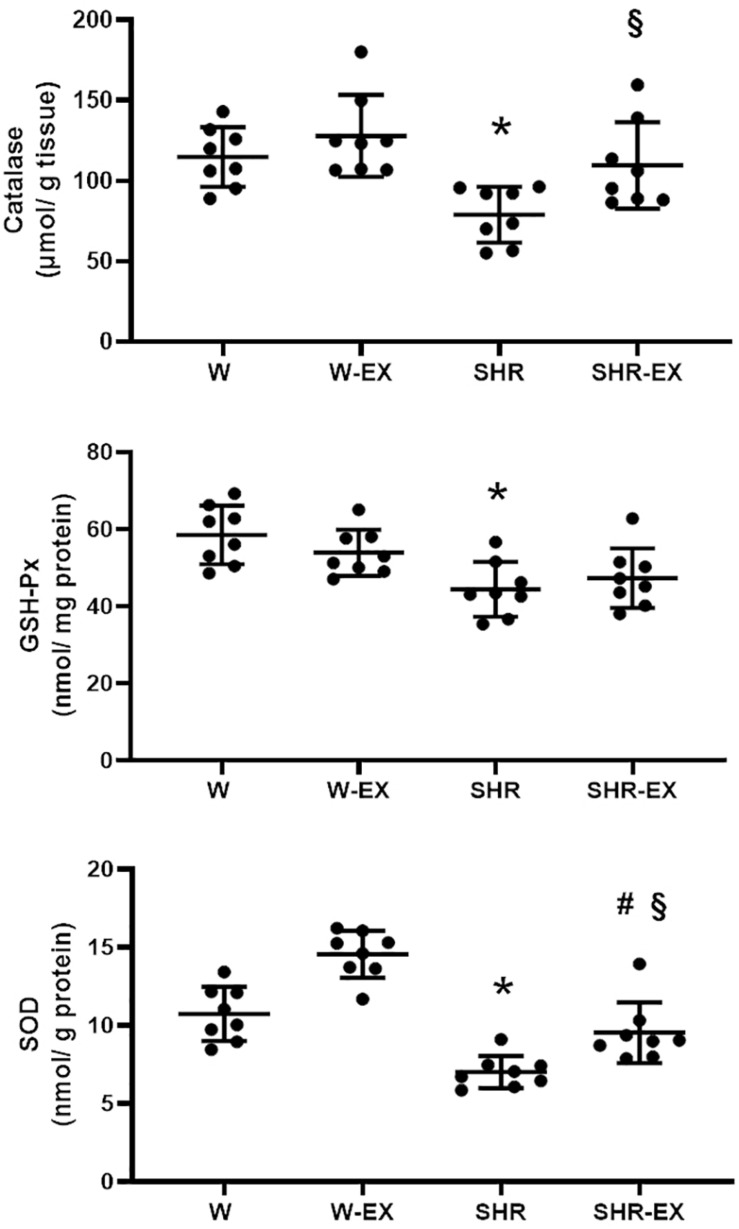
Myocardial antioxidant enzyme activities. GSH-Px: glutathione peroxidase; SOD: superoxide dismutase. W, Wistar rats; W-EX, exercised Wistar rats; SHR, spontaneously hypertensive rats; SHR-EX, exercised SHR. ANOVA for a 2 × 2 factorial design and Tukey; *p < 0.05 vs. W; #*p* < 0.05 vs. W-EX, §*p* < 0.05 vs. SHR; *n* = 8 for all analyzes.

Myocardial antioxidant enzyme activities are shown in Figure 2. SHR had lower enzyme activity than W and lower CAT and SOD activity than SHR-EX. SHR-EX presented lower SOD activity than W-EX.

Serum and myocardial malondialdehyde concentration and NADPH oxidase activity did not differ between groups (Table 7). Myocardial lipid hydroperoxide was higher in both SHR groups than their respective controls. SHR had lower β-hydroxyacyl dehydrogenase and higher lactate dehydrogenase activities than W. SHR-EX presented higher citrate synthase and β-hydroxyacyl dehydrogenase and lower lactate dehydrogenase activity than SHR.

**TABLE 7:** Oxidative stress and energy metabolism.

	W (*n* = 8)	W-EX (*n* = 8)	SHR (*n* = 8)	SHR-EX (*n* = 8)
Serum MDA (μM/L)	1.83 ± 0.39	1.74 ± 0.37	1.86 ± 0.42	2.01 ± 0.74
Myocardial MDA (nmol/mg protein	0.86 ± 0.12	0.70 ± 0.06	0.87 ± 0.22	0.76 ± 0.25
Lip. Hydrop. (nmol/g tissue)	203 ± 33	183 ± 26	248 ± 24*	226 ± 38^#^
NADPH oxidase activity (x10^6^ UA/mg protein)	3.42 ± 1.4	2.65 ± 1.45	2.50 ± 0.98	2.87 ± 0.70
Citrate synthase (nmoL/mg protein)	23.8 ± 4.26	26.9 ± 4.96	19.8 ± 3.24	30.0 ± 6.14^§^
β-HAD (nmoL/mg protein)	38.5 ± 7.20	36.4 ± 8.36	25.7 ± 5.68*	36.8 ± 7.52^§^
Lactate dehydrogenase (nmol/mg protein)	89.5 ± 8.79	81.2 ± 11.2	123 ± 18.7*	89.2 ± 13.6^§^

Data as mean ± standard deviation. W, Wistar rats; W-EX, exercised Wistar rats; SHR, spontaneously hypertensive rats; SHR-EX, exercised SHR; MDA, malondialdehyde concentration; Lip. hydrop., lipid hydroperoxide concentration; β-HAD, β-hydroxyacyl dehydrogenase. ANOVA for a 2 × 2 factorial design and Tukey; *p < 0.05 vs. W; #*p* < 0.05 vs. W-EX; §*p* < 0.05 vs. SHR.

**FIGURE 3:**
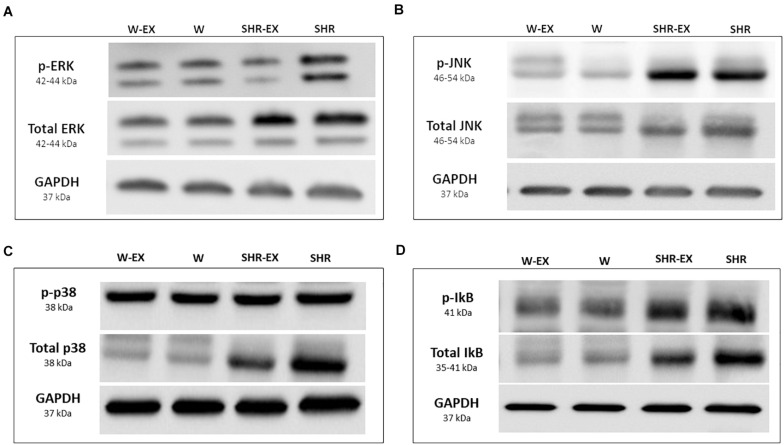
Representative Western blots. W, Wistar rats; W-EX, exercised Wistar rats; SHR, spontaneously hypertensive rats; SHR-EX, exercised SHR; **(A)** ERK, extracellular-signal-regulated kinase; **(B)** JNK, Jun N-terminal kinase; **(C)** p-38; **(D)** IκB, inhibitor of nuclear factor kappa B.

Table 8 shows protein expression and Figure 3 shows representative western blots. Phosphorylated JNK, total p38, phosphorylated and total ERK, and total IκB were higher in SHR than W. Phosphorylated JNK and total IκB were higher in SHR-EX than W-EX, and phosphorylated ERK was lower in SHR-EX than SHR.

**TABLE 8:** Protein expression.

		W (*n* = 7)	W-EX (*n* = 7)	SHR (*n* = 7)	SHR-EX (*n* = 7)
JNK	Phosphorylated	1.00 ± 0.31	1.02 ± 0.31	3.46 ± 1.23*	3.38 ± 1.61^#^
	Total	1.00 ± 0.47	0.91 ± 0.40	1.54 ± 0.59	1.09 ± 0.27
p38	Phosphorylated	1.00 ± 0.37	0.85 ± 0.27	1.10 ± 0.18	1.17 ± 0.49
	Total	1.00 ± 0.22	0.76 ± 0.22	3.38 ± 1.79*	3.36 ± 2.08
ERK	Phosphorylated	1.14 (0.69–1.21)	1.14 (0.99–1.44)	6.03 (2.08–10.8)*	0.90 (0.26–0.99)^§^
	Total	1.00 ± 0.41	0.66 ± 0.24	1.54 ± 0.44*	1.12 ± 0.55
p65 NF-kB	Phosphorylated	1.00 ± 0.69	0.96 ± 0.45	1.03 ± 0.49	0.63 ± 0.60
	Total	1.00 ± 0.50	0.61 ± 0.17	0.90 ± 0.62	0.90 ± 0.88
IκB	Phosphorylated	1.00 ± 0.67	0.75 ± 0.38	1.53 ± 0.73	1.01 ± 0.85
	Total	1.00 ± 0.67	0.67 ± 0.26	3.67 ± 1.50*	2.57 ± 1.56^#^

Data as mean ± standard deviation or median and percentiles. W, Wistar rats; W-EX, exercised Wistar rats; SHR, spontaneously hypertensive rats; SHR-EX, exercised SHR; JNK, Jun N-terminal kinase; ERK, extracellular-signal-regulated kinase; p65 NF-κB, p65 subunit of nuclear factor kappa B; IκB, Inhibitor of nuclear factor kappa B. ANOVA for a 2 × 2 factorial design and Tukey or Kruskal–Wallis and Dunn; *p < 0.05 vs. W; #**p** < 0.05 vs. W-EX; §**p** < 0.05 vs. SHR.

## Discussion

Spontaneously hypertensive rats develop arterial hypertension at approximately 2-month age and initiate LV hypertrophy at 3-month age (Bing et al., 1995). The rats then present compensated hypertrophy with preserved cardiac function for a long period (Oliveira Junior et al., 2010). Between 18 and 24 months of age, the animals develop ventricular dysfunction and heart failure evolving to death within a few weeks after cardiac decompensation (Damatto et al., 2016). Therefore, to avoid a high mortality rate, we started physical exercise at the age of 13 months.

Several exercise tests have been used to assess physical capacity and prescribe exercise intensity. Considering maximum intensity tests, different protocols have been applied in rodents, such as incremental test, submaximal test, and constant speed test (Abreu et al., 2012; Almeida et al., 2012). A reliable test is the measurement of maximal oxygen consumption (VO_2_ max), which evaluates oxygen consumption; however, besides being expensive, it requires a spirometer to be performed (Rodrigues et al., 2007). Maximal lactate steady state (MLSS) characterizes the maximum exercise intensity in which blood lactate concentration remains constant (Ferreira et al., 2007). It has been used to distinguish the limits of metabolic domains by identifying the transition between aerobic and anaerobic metabolism (Abreu et al., 2016; Lonbro et al., 2019). Although MLSS is useful in an individual basis, it is little feasible when working with groups of rodents. Therefore, it is important to choose the test which fits better the experimental model (Abreu et al., 2016). To establish the training protocol, we used the maximum effort test, which consists of a subjective assessment of the maximum capacity to run in a graded treadmill. Our exercise protocol was based in previous studies with aged SHR (Emter et al., 2005; Chicco et al., 2008; Pagan et al., 2015). We used a low-intensity aerobic protocol because it was well tolerated, safe, and effective in SHR at an advanced stage of uncontrolled arterial hypertension (Pagan et al., 2015). On the other hand, a high-intensity protocol was associated with worsening cardiac remodeling and accelerated progression to heart failure in elderly SHR (Schultz et al., 2007; da Costa Rebelo et al., 2012). It is therefore probable that a high intensity exercise program would have produced deleterious cardiac effects. Although SHR performed better in the initial test than the Wistar, the protocol was started with a low intensity for all animals. A probable consequence of the low intensity protocol was that we could not find any differences between W-EX and W groups, except for improved functional capacity. On the other hand, the low intensity protocol had beneficial effects on heart failure development as heart failure features were lower in SHR-EX than SHR. We evaluated myocardial function, oxidative stress markers, and energy metabolism as probable mechanisms involved in heart failure attenuation.

Myocardial function was analyzed in isolated LV papillary muscle preparations, which allows measurement of myocardial contractility regardless of changes in cardiac load, heart rate, and ventricular chamber geometry. Both SHR groups had higher RT and -dT/dt than their respective controls. RT is mainly related to the myocardial collagen content (Matsubara et al., 1997), which is increased in the senescent SHR (Cezar et al., 2015; Pagan et al., 2015, 2019). The –dT/dt values are dependent on the diastolic Ca^2+^ removal from cytoplasm. Main factors involved in diastolic Ca^2+^ transient are Ca^2+^ uptake by the sarcoplasmic reticulum (SR) through SR Ca^2+^-ATPase 2 (SERCA2), and Ca^2+^ extrusion from myocyte (Bers, 2002). During compensated myocardial hypertrophy, a higher SERCA2 activation may increase SR Ca^+2^ uptake and improve active relaxation ^45^. Our data therefore show that 18-month old SHR present impaired myocardial passive property with better active relaxation.

Inotropic stimulation has been used to identify changes in cardiac muscle contraction and relaxation that cannot be observed at basal condition. In the baseline state, papillary muscles from SHR-EX had a better contractile performance than W-EX, and higher +dT/dt than SHR. After inotropic stimulation, both SHR and SHR-EX displayed better function than their controls, which was more evident in SHR-EX. SHR papillary muscles present preserved or improved mechanical function until approximately 18 month-old; only in senescence, does cardiac function to deteriorate (Bing et al., 1988; Cezar et al., 2013). Increased myosin adenosine triphosphatase activity and improved calcium transient may be involved in the improved response to isoproterenol (Bers, 2002; MacDonnell et al., 2005; Pinter et al., 2008). Exercise-induced improvement in myocardial function has been described in hypertensive rats at both basal condition and after inotropic stimulation (Fernandes et al., 2015). Trained SHR had improved inotropic responsiveness to β-adrenergic stimulation and increased phosphorylation of calcium transient proteins (MacDonnell et al., 2005). Thus, our data show that exercise is associated with myocardial functional benefit in hypertensive rats.

Oxidative stress is related to the pathophysiology of arterial hypertension; an imbalance between ROS production and antioxidant capacity induces vascular dysfunction and increases blood pressure (Montezano et al., 2015; Larsen and Matchkov, 2016). Malondialdehyde and lipid hydroperoxide were assessed as oxidative stress markers. Both hypertensive groups showed increased myocardial lipid hydroperoxide concentrations, which were not changed by exercise. Another mechanism involved in oxidative stress is the NADPH oxidase complex, an important source of ROS generation (Touyz et al., 2020). NADPH oxidase activity did not differ between groups, suggesting that it was not involved in the increased oxidative stress in the hypertensive groups.

Increased ROS production activates antioxidant defense mechanisms. To evaluate antioxidant capacity, we assessed myocardial CAT, SOD, and GSH-Px activity. The improved CAT and SOD activity in SHR-EX compared to SHR shows that exercise increased antioxidant capacity but was not effective in reducing myocardial oxidative stress. Previous studies have shown reduced lipid peroxidation with increased antioxidant capacity in young exercised hypertensive animals (Bertagnolli et al., 2008; da Palma et al., 2016). A single exercise session increases ROS production; however, following chronic physical training, the increased antioxidant defense reduces oxidative stress in healthy animals (Fisher-Wellman and Bloomer, 2009; Gomes et al., 2017). Our results suggest that in long-term hypertensive rats, chronic exercise is not associated with reduced oxidative stress, despite increased antioxidant capacity.

Increased oxidative stress activates the MAPK signaling pathway which is involved in cardiac hypertrophy and heart failure. This pathway, also activated by extracellular stimuli including angiotensin II, endothelin-1, noradrenaline, and growth factors, induces cellular growth, apoptosis, and necrosis (Marber et al., 2011). It is possible that other stimuli besides lipid hydroperoxide were involved in activating MAPK in our hypertensive groups, such as increased sympathetic nervous system activity and the post-load-induced increase in LV wall stress (Richer et al., 1997). The main kinases of this pathway are ERK, JNK and p38 (Wang, 2007). ERK catalyzes phosphorylation of transcription factors that stimulate protein synthesis and cell growth, whereas JNK and p38 modulate myocardial apoptosis, synthesis of inflammatory cytokines, and fibrosis (Zhang et al., 2003). Our results showed increased total ERK and p38, and increased phosphorylation of JNK and ERK in SHR. In the SHR-EX, restoration of phosphorylated and total ERK was seen, while phosphorylated JNK remained higher than W-EX. Blockade of p38 activity has been associated with reduced LV hypertrophy and dysfunction in SHR (Behr et al., 2001).

NF-κB is a transcription protein that regulates inflammatory cascade genes (Lawrence, 2009). NF-κB is a heterodimer consisting of two subunits: p65 and p50. When not stimulated, NF-κB is in the cytoplasm connected to an inhibitory protein, IkB. This complex prevents translocation of NF-κB to the nucleus. Phosphorylation of IkB releases NF-κB, which acts on target genes in the nucleus while IkB is degraded (Baeuerle et al., 1988). We observed that the p65 subunit did not differ between groups, and total IkB was increased in the hypertensive groups with no influence of the physical exercise. The increase in IkB has probably normalized the expression of the p65 NF-κB in SHR-EX, suggesting that increased IkB inhibited activation of genes involved in myocardial inflammation and hypertrophy. Although NF-κB can be activated by the MAPK ERK and p38, the increase of these proteins did not change NF-κB activation in the SHR group.

Uncontrolled and long-term hypertension may change the use of substrate for myocardial energy production (Christe and Rodgers, 1994). The heart alters the preferred use of fatty acids for glucose. In addition, a decreased oxidative capacity and energy production, and a reduced energy transfer by phosphotransferases lead to a decreased efficiency of energy consumption (Ventura-Clapier, 2009). Our data suggest that the use of fatty acids as the main energy substrate, changed in SHR, was preserved in SHR-EX, which presented unchanged β-hydroxyacyl dehydrogenase, lactate dehydrogenase and citrate synthase activities. Thus our data suggest that exercise-induced normalization of energy metabolism and increase in antioxidant enzyme activity may have attenuated ERK phosphorylation and contributed to the better functional capacity and myocardial function in SHR-EX.

A limitation of this study is that we have used Wistar instead of Wistar-Kyoto rats. As SHR were originated from Wistar-Kyoto rats, this strain has been used as a genetic control for SHR.

In conclusion, physical exercise improves physical capacity and myocardial function and reduces the frequency of heart failure features in SHR. In addition, exercise increases antioxidant enzyme activity, decreases ERK phosphorylation and lactate dehydrogenase, and attenuates total ERK protein expression.

## Data Availability Statement

The original contributions presented in the study are included in the article/supplementary material, further inquiries can be directed to the corresponding author/s.

## Ethics Statement

The animal study was reviewed and approved by Botucatu Medical School Animal Experimentation Ethics Committee, UNESP, São Paulo, Brazil.

## Author Contributions

LP and KO: conception and design of study, acquisition of data, analysis and interpretation of data, and manuscript writing. MG: acquisition of data and interpretation of data. RD, ARL, MC, FD, DR, DC, and TC: data collection. BP, DF, FL, AF, AL, and AC: data collection and analysis. MO: manuscript writing. All authors contributed to the article and approved the submitted version.

## Conflict of Interest

The authors declare that the research was conducted in the absence of any commercial or financial relationships that could be construed as a potential conflict of interest.
